# Injury to the Phrenic Nerve

**Published:** 1890-02-08

**Authors:** 


### RECENT RESEARCH.

### INJURY TO THE PHRENIC NERVE.

Some time ago we noticed a case reported by Dr. Coull Mackenzie, Health Officer, Calcutta, of sudden death attributed to rupture of the phrenic nerve; this being the only injury discovered on a *post mortem* examination. It was then observed that little was really known with regard to injuries of the phrenic nerve, and we felt rather disposed to attribute the sudden death to shock than to the rupture of the one phrenic nerve, as breathing is not carried on entirely by the diaphragm. Quite recently, Dr. H. A. Hare and Dr. E. Martin, Professors in the Pennsylvania University, have investigated the effect of injuries of the phrenic nerve. It has usually been taught that the chief function of the phrenic nerve is to innervate the diaphragm, which is, indeed, the fact. But it has also been taught that, if the phrenic nerves are in any way cut or broken, the diaphragm becomes but a relaxed and flabby piece of muscle, and thereby is rendered unable to fulfil its function. This also may be accepted; but it does not follow that death must result, because the diaphragm does not act. Dr. Hare and Dr. Martin correctly observe: "In the human being, whose respiratory movements are largely thoracic, logical reasoning brings us to the conclusion that injuries to the phrenic nerve should not be certainly fatal." The experimentalists found that, in some of the higher animals whose breathing is largely thoracic, no untoward effects followed phrenic section. It would, therefore, appear that the cause of death in the Calcutta case could not have been injury to the phrenic nerve, although such conclusion was justified by the teaching of the schools. It may be mentioned that Dr. Hare and Dr. Martin say that the diaphragm plays a much more important part in respiration in the male than in the female.

